# Development of a Diagnostic Biosensor Method of Hypersensitivity Pneumonitis towards a Point-of-Care Biosensor

**DOI:** 10.3390/bios11060196

**Published:** 2021-06-15

**Authors:** Tatiana Fiordelisio, Ivette Buendia-Roldan, Mathieu Hautefeuille, Diana Del-Rio, Diana G. Ríos-López, Diego Zamarrón-Hernández, Samuel Amat-Shapiro, Andrea Campa-Higareda, Edgar Jiménez-Díaz, Erika González-Villa, Janikua Nelson-Mora, Natllely García-Carreño, Jehú López-Aparicio, Eduardo Montes, Armando Santiago-Ruiz, Annie Pardo, Moisés Selman

**Affiliations:** 1 Departamento de Biología, Facultad de Ciencias, Universidad Nacional Autónoma de México, Mexico City 04510, Mexico; delrio@ciencias.unam.mx (D.D.-R.); edgarjd@ciencias.unam.mx (E.J.-D.); annie.pardo@ciencias.unam.mx (A.P.); 2 Laboratorio Nacional de Soluciones Biomiméticas para Diagnóstico y Terapia LANSBioDyT, Universidad Nacional Autónoma de México, Mexico City 04510, Mexico; mathieu_h@ciencias.unam.mx (M.H.); grisel@ciencias.unam.mx (D.G.R.-L.); dgzmhr@ciencias.unam.mx (D.Z.-H.); samuel_amat@ciencias.unam.mx (S.A.-S.); gdmcrandy@hotmail.com (A.C.-H.); erika.gv@ciencias.unam.mx (E.G.-V.); janikuanelson@ciencias.unam.mx (J.N.-M.); natllely.garcia@ciencias.unam.mx (N.G.-C.); jehu@ciencias.unam.mx (J.L.-A.); mselmanl@yahoo.com.mx (M.S.); 3 Instituto Nacional de Enfermedades Respiratorias Dr. Ismael Cosio Villegas, Mexico City 14080, Mexico; ivettebu@yahoo.com.mx (I.B.-R.); eduardomontesm@gmail.com (E.M.); santiagoarmando881@gmail.com (A.S.-R.); 4 Departamento de Física, Facultad de Ciencias, Universidad Nacional Autónoma de México, Mexico City 04510, Mexico

**Keywords:** magnetic beads, antibodies, PoC

## Abstract

In spite of a current increasing trend in the development of miniaturized, standalone point-of-care (PoC) biosensing platforms in the literature, the actual implementation of such systems in the field is far from being a reality although deeply needed. In the particular case of the population screenings for local or regional diseases related to specific pathogens, the diagnosis of the presence of specific antibodies could drastically modify therapies and even the organization of public policies. The aim of this work was to develop a fast, cost-effective detection method based on the manipulation of functionalized magnetic beads for an efficient diagnosis of hypersensitivity pneumonitis (HP), looking for the presence of anti-pigeon antigen antibodies (APAA) in a patient’s serum. We presented a Diagnostic Biosensor Method (DBM) in detail, with validation by comparison with a traditional high-throughput platform (ELISA assay). We also demonstrated that it was compatible with a microfluidic chip that could be eventually incorporated into a PoC for easy and broad deployment using portable optical detectors. After standardization of the different reaction steps, we constructed and validated a plastic chip that could easily be scaled to high-volume manufacturing in the future. The solution proved comparable to conventional ELISA assays traditionally performed by the clinicians in their laboratory and should be compatible with other antibody detection directly from patient samples.

## 1. Introduction

Hypersensitivity pneumonitis (HP) is a complex syndrome caused by exposure to a large variety of organic particles, small enough (~5 µm) to reach pulmonary alveoli [1,2]. HP is an immune-mediated interstitial lung disease occurring in susceptible individuals [1,2,3]. Importantly, in a number of cases, HP can lead to pulmonary fibrosis, which is associated with progressive respiratory insufficiency and death. Exposure may occur indoors, at work, at home, or even outdoors in recreational environments. In Mexico and many other countries, the main disease-associated exposure is contact with pigeons and domestic birds [4]. Key actions in HP treatment consist in early diagnosis and antigen avoidance, as it may also allow limiting an aggressive progression of the disease in affected patients [5]. Indeed, disease remission is possible in patients with an inflammatory, non-fibrotic disease with early diagnosis, antigen avoidance, and 3–6 months of treatment [1]. In most cases, however, patients need to be treated with immunosuppressive drugs for a long time [2]. Prolonged treatment and death occur mainly if the antigens and their source are not identified [2,6].

The diagnosis of HP requires the integration of multiple domains, including radiological, functional, and bronchoscopic/histopathological findings, but is particularly challenging in the absence of an identifiable exposure. In this context, antigen identification is critical to tackling HP. Measurement of specific immunoglobulin G antibodies (IgG) in a patient’s blood serum is an important method used to identify an inciting antigen and is proposed as a diagnostic test for HP [7,8,9]. There are several techniques to detect specific IgG in blood serum, the enzyme-linked immunosorbent assay (ELISA) being the most used thanks to its reproducibility, specificity, and sensitivity [10].

Recent combined progress in microtechnology and precise protein immobilization on a variety of materials allowed for the development of microfluidic platforms designed as low cost easily operated point-of-care (PoC) biosensing systems offering a broader availability in locations where the use of costly and complex instruments is not an option [11,12,13]. 

Compared with more conventional existing techniques, these PoC systems can integrate high-sensitivity detection methods with simple readouts and data logging platforms to provide rapid and cost-effective solutions for long-term monitoring and diagnostics [14,15]. Large population screenings were also possible in some cases, ideal for low-income and remote areas, without specialized medical centers or limited access to laboratories and facilities [16,17,18]. Miniaturization of ELISA sensors was also achieved using microtechnology, making it a very promising family of detection techniques in the near future thanks to a simplification of readouts and a decrease in costs [19,20,21]. However, in this particular case of immunoassays biosensors, the integration towards PoC solutions was still limited by the need for several washing steps on-chip that implied a tedious manipulation of low-volume samples inside plastic microfluidic channels. More recent works then focused on improvements or modifications of the current detection method by the use of nanomaterials or complex measurement techniques that rather complicated the development and use and, hence, limited their current implementation [22,23,24,25]. 

One of the possible simple solutions to integrate ELISA platforms in microfluidics chips that may help to overcome these restrictions consists in the use of magnetic beads [22]. Their small size and spherical geometry allow them to precisely and reproducibly immobilize all sorts of biomolecules, increasing the sensitivity of optical or amperometric immunoassays [26,27,28]. Thanks to their reaction surface and their simple contactless manipulation using magnets, it has been possible to reduce reaction times, buffer, and antibody solution volumes [22]. These advantages also helped reduce the cost of each test in comparison with current ELISA methods, and magnetic beads have successfully been employed to improve the development of patients’ antibody detection in PoC platforms [29,30,31].

The aim of this work was to design a biosensing technique compatible with a simple, low-cost PoC platform to detect the presence of human IgG antibodies in patient’s serum samples of avian-antigen-induced HP, using pigeon antigen sera coupled to commercial magnetic beads. After validation of all the different reactions of the method in microwells at the lab bench level, a final microfluidic chip was designed and tested for future use in PoC. For this purpose, a motorized actuated platform was also fabricated to precisely manipulate functionalized magnetic beads throughout biological samples and to bind specifically the desired antibodies inside a microfluidic chip that allowed a simple optical readout in an ELISA-like fashion. The method, called Diagnostic Biosensor Method (DBM) in all the following, was successfully validated using patient’s samples, and preliminary results compared well with a traditional ELISA performed in a hospital clinical laboratory, which is currently used to diagnose HP.

## 2. Materials and Methods

### 2.1. Serum Samples

We used samples from 30 HP patients of the National Institute of Respiratory Diseases (INER). HP was diagnosed as previously described, using clinical symptoms, history of antigen exposure, and specific antibodies against avian proteins measured by conventional ELISA, pulmonary function tests, high-resolution computed tomography, an increase in lymphocytes in bronchoalveolar lavage, and/or surgical biopsy [1,2]. The serum was obtained from venous blood by the medical doctors for the detection of human IgG anti-pigeon-dropping extract. The volunteers gave informed consent, and the study was approved by the Ethics Committee of INER, with number C07-19. Serum samples were stored at −20 °C until use. Protein concentration of control HP-positive patient serum sample, 110 μg/μL, was obtained by the Protein A280 method using a full spectrum UV visible microvolume spectrometer (NanoDrop 2000, Thermo Scientific, Wilmington, DE, USA). Serum samples from normal individuals without known exposure and without antibodies for avian antigens were used as negative controls.

### 2.2. Pigeon Antigen Production

The method used in this work followed a published procedure [32] in which a pool of 10 pigeon sera (PS) was obtained by cardiac puncture and used as a source of antigens. The protein concentration of this pool, as determined by Lowry’s method, was 30 mg/mL. PS was aliquoted and stored at −70 °C until used.

### 2.3. ELISA

The conventional ELISA was performed with a microtiter plate coated with the pigeon sera diluted in a carbonate buffer, incubated overnight at 4 °C, and washed 3 times with PBS-Tween 20. Plates were then blocked with PBS-Tween containing 0.5% bovine serum albumin (BSA) for 1 h at 37 °C on a horizontal orbital microplate shaker and washed 3 times with PBS-Tween 20. Patient sera were diluted 1:6250 for the IgG assay in PBS-Tween containing 0.5% BSA. Under these conditions, each sample was studied in triplicate, and plates were incubated for 1 h at 37 °C with shaking. Purified goat anti-human IgG (Fc specific) conjugated with horseradish peroxidase (Calbiochem, Darmstadt, Germany) was added and incubated for 1 h at 37 °C with shaking. Finally, orthophenylenediamine (Sigma Aldrich, Steinheim, Germany) was added and incubated at 37 °C with shaking for 10 min, and the reaction was stopped with H_2_SO_4_ 1 mol/L. The absorbance was read in a microplate spectrophotometer at 492 nm with a correction factor at 595 nm.

Serum samples were measured simultaneously using conventional ELISA assay versus the detection with the developed method of magnetic beads. 

### 2.4. Diagnostic Biosensor Method of Avian-Related Hypersensitivity Pneumonitis

**Figure 1:**
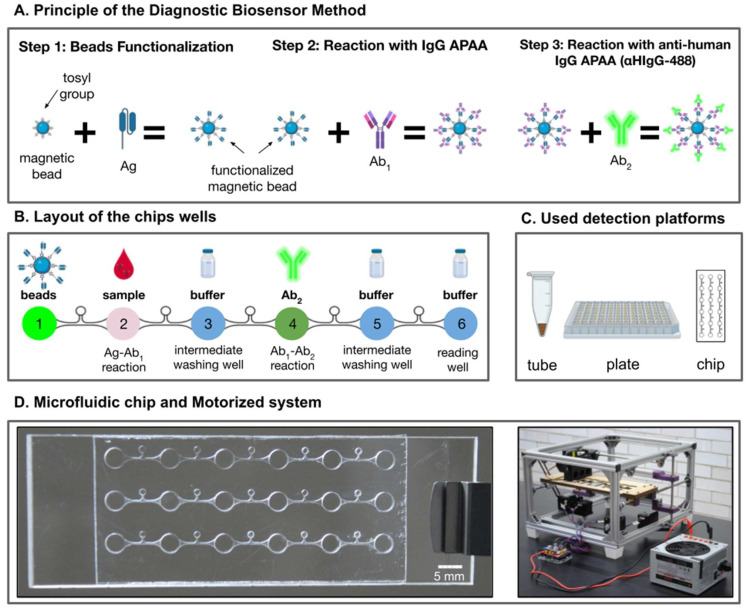
The schematization of the steps of the Diagnostic Biosensor Method. (**A**) Preparation and reactions used in the method; (**B**) details of the detection sequence followed inside the microfluidic chip; (**C**) schemes of the detection platforms used in this project. Ag: antigen, Ab1: primary antibody, Ab2: secondary antibody; (**D**) photographs of the chip design and the motorized platform used to control beads movement.

The Diagnostic Biosensor Method presented here was based on the use of functionalized magnetic beads as elements of biological recognition to detect biomolecules of interest contained in a sample using an immunodetection method. The detection procedure consisted of three steps, depicted in Figure 1A. To apply the DBM to the detection of IgG anti-pigeon antigen antibodies (APAA), the magnetic beads were functionalized with PS, containing pigeon antigens. Then, the beads functionalized with pigeon antigens reacted with a patient serum sample (with an unknown concentration of APAA, to be detected). Finally, the beads reacted with fluorescent conjugated anti-human IgG secondary antibody (αHIgG-488). This secondary antibody (Ab_2_) reaction was employed for optical detection by fluorescence microscopy and quantification of the signal emitted by the Ab_2_-marked beads.

This DBM was versatile and could be used in either reaction microtubes, 96 well plates, as well as a transparent microfluidic chip. All the reactions were first standardized and optimized in 1.5 mL microtubes and non-treated polystyrene flat-96-well assay plates prior to the final tests inside the system of the microfluidic chip. This diagnostics validation with patient samples was performed inside ad hoc microfluidic chips (see Figure 1 and Supplementary Information Figure S1.1). 

Although different concentrations of magnetics beads and volumes of reaction solutions were used, the same ratio of proteins (PS, APAA, and αHIgG-488), with respect to the number of beads, was maintained in each test platform. We herein related all the protocol steps to 80 K magnetic beads, number of beads used in the microfluidic chip (80 K beads in a 0.2 µL volume, up to a maximum of 12 µL).

#### 2.4.1. Step 1: Beads Functionalization

The beads functionalization consisted of a covalent coupling reaction between the amino and sulfhydryl functional groups of the pigeon antigen and the tosyl groups of magnetic beads (Dynabeads^®^ Tosylactivated M-450 Invitrogen, Carlsbad, CA, USA), basing the protocol on the manufacturer’s recommendations. Beads were resuspended by vortexing (Vortex Mixer, DLAB, Beijing, China); the desired amount of beads was transferred to a 1.5 mL low binding microtube (T4816-250EA, Sigma-Aldrich, Darmstadt, Germany) and washed with 500 μL of Buffer 1 (sodium phosphate buffer: NaH_2_PO_4_ 0.1 mol/L, Na_2_HPO_4_ 0.1 mol/L at pH = 8; S0751 and S0876, Sigma-Aldrich, Darmstadt, Germany). Then, beads were resuspended in 500 µL of a PS solution in Buffer 1 (tested concentrations: 0, 0.4, 0.5, 0.6, 0.8, 1, 1.5, 3 μg/80 K beads) and it was incubated for 24 h (16 h and 24 h were tested) at room temperature while shaking in a HulaMixer (HulaMixer Sample Mixer, Invitrogen, Carlsbad, USA) setting orbital 15 rpm/10 s; reciprocal 20°/15 s; vibro 1°/1 s, horizontal, allowing tilt and rotation of microtubes. Afterward, two washes of 5 min each were performed with 500 µL of Buffer 2 at 2–8 °C (PBS 0.1 mol/L Ca^2+^ and Mg^2 +^ free, BSA 0.1% supplemented, and EDTA 2 mmol/L; pH = 7.4; D5652, A2153, and E5134; Sigma Aldrich, Darmstadt, Germany) with a HulaMixer agitation. In order to block the remnant tosyl groups, beads were then resuspended with 500 μL of Buffer 3 (Tris base 0.2 mol/L, BSA 0.1% supplemented; pH = 8.5; T1503 and A2153 Sigma-Aldrich, Darmstadt, Germany) and incubated for 4 h in Hulamixer agitation at 37 °C (222DS, LabNet, Edison, New Jersey, USA). After blocking the incubation, a final wash with Buffer 2 was performed, as described above. The beads were resuspended in a vortex (30 s) at the desired volume (10 µL/80 K beads) in PBS supplemented with 0.01% Tween at pH = 7.4 (PBS-Tween; D5652 and P9416; Sigma Aldrich, Darmstadt, Germany) and immediately transferred (10 μL) to the corresponding reaction platform. As described in Supplementary Information S1.1c, this surfactant was added to increase the wettability of the beads surface area, therefore, improving the mobility of the beads in the microfluidic chip (the PBS used in the reactions carried out in 1.5 mL low binding microtubes was not supplemented with Tween).

#### 2.4.2. Step 2: Reaction with IgG Anti-Pigeon Antigen Antibodies (APAA)

In this stage, to standardize the DBM and determine its sensitivity, we used a dilution curve of a control (positive) sample with different APAA concentrations (2.8, 4.6, 6.1, 8.8, 18.3, 36.7, 110 μg/80 K beads). For all the samples with unknown concentrations, 0.08 μL patient serum/80 K beads were used for diagnostic probes. The reaction was carried out on the different platforms as follows. Inside the chips, the beads were displaced using a motorized actuator platform (described in Supplementary Information S1.2 and Figure S1.2.1) towards the patient sample solution well (in PBS-Tween 0.01%, 10 μL) passing through the intermediate washing well. In microtubes and plates, after the washing step, the beads were agglomerated using magnets, the supernatant was discarded, patient and negative serum solution prepared in PBS were added (microtubes 500 μL; plate 25 μL) and resuspended by the vortex. Different incubation times were tested: 15, 45, and 120 min at room temperature while shaking (microtubes in HulaMixer, 96-plate in orbital shaker 100 rpm and in chip, motorized actuator platform; see Section 2.5.3). As a negative control, the functionalized beads reacted with PBS or PBS-Tween 0.01% instead of APAA from the patient’s serum, and later with αHgG-488.

Furthermore, thanks to the design of the microfluidic chip and the motorized platform, the washing steps with a grouping of beads with magnets and supernatant removal (microtube, by aspiration; plate, by decantation) were eliminated on the chip: instead, to minimize the hauling of undesired free antibodies caused by the dragging force of the beads during their movement from one well to another, the chip design included intermediate wells with PBS-Tween 0.01% (Figure 1).

#### 2.4.3. Step 3: Reaction with Anti-Human IgG APAA (αHIgG-488)

Finally, to detect and quantify the bounded IgG APAA, a reaction with Alexa Fluor^®^ 488-conjugated AffiniPure Alpaca Anti-Human IgG (H + L) secondary antibody (RRID:AB_2721859; Jackson ImmunoResearch Labs Cat# 609-545-213, Lot# 135869, 1 mg/mL, West Grove, USA; αHIgG-488) was performed, and different concentrations were tested to determine the optimal one (0, 0.5, 0.6, 1.3, 1.7, 2.5 μg/80 K beads). The procedure ran as follows: in a chip, the beads were mobilized to the αHIgG-488 well (10 μL; PBS Tween 0.01%); in microtubes and plate αHIgG-488 solution was added (microtubes 500 μL; plate 25 μL; PBS) and resuspended by the vortex. Incubated (tested: 15, 45, and 120 min) at room temperature while shaking (chip, motorized actuator platform; microtubes, HulaMixer; plate, orbital shaker 100 rpm). Inside the chip, the beads were just mobilized to the optical reading well (PBS-Tween 0.01%, 10 μL), passing through the intermediate washing well. In microtubes and plates, PBS washes were performed as described in Step 2, and the beads were resuspended in PBS (microtubes 10 μL; plates 40 μL) for the final optical readout. 

#### 2.4.4. Confocal Microscopy Detection and Image Analysis

The magnetic beads processed in microtubes and plates were observed with the epifluorescence microscope TCS-SP8 (LEICA, Wetzlar, Germany). After mixing the sample, a 10 μL drop was placed on a slide, and a magnet was used to spread the beads on a single focal plane. The beads were excited at 488 nm, with the GFP filter (Ex. BP 470/40, DM 500, Em. BP. 525/50). To visualize the entire sample, a 10× magnification was used with an exposure time of 4 s and a gain of 10. The final xy resolution was 1.118 µm. Several images per sample were taken and exported in tiff format for analysis.

#### 2.4.5. Image Processing

Fluorescence intensity was calculated per sample through the computing of the fluorescence intensity at three different levels: bead level, image level, and sample level. At the bead level, a Laplacian of Gaussian filter (LoG filter) was used for the beads segmentation and detection in the images. After precise spatial location, the fluorescence intensity of each bead was calculated by averaging the values of the set of pixels constituting it. The minimum and maximum intensity levels, the number of identified beads, and the average fluorescence intensity per image was obtained. Besides the average, two additional values were computed: the 75th percentile and the sum of all N values above the 25th percentile divided by N (the weighted sum). Finally, the fluorescence intensity values of all the images related to a single sample were used to compute the same values (average fluorescence intensity, average of the 75th percentile, and weighted sum). In all averaging cases, an associated standard deviation was computed, including data used for the weighted sum. A detailed description of the method used to detect the beads in the images was discussed in the Supplementary Information S2.

### 2.5. Microfluidic Chip

#### 2.5.1. Fabrication

An ad hoc microfluidic chip with a motorized actuated system was conceived to precisely manipulate magnetic beads with magnets inside microwells. It could be regarded as the first functional prototype, a promising progress towards a standalone reader and actuator for PoC implementation. The motorized system was constructed with aluminum extrusion, stepper motors, microcontrollers (Arduino and Teensy), laser cut, and 3D printed custom parts; all of these materials were readily available and relatively inexpensive. The final design and fabrication procedure were set after a careful standardization of bead displacement and washing steps that was described in the Supplementary Information S1. The layout of the chip consisted of microfluidic channels specially designed to connect consecutive wells made for different purposes (Figure 1B and Supplementary Information Figure S1.1) and between which no mixing was permitted during the displacement of the beads to avoid cross-contamination. The layout was designed with Computer-Aided Design (CAD) tools and fabricated using a micro-milling machine (CNC Mini-Mill/4, Minitech Machinery Corp., Norcross, GA, USA), a subtractive manufacturing technique [33], on which a milling tip with a 254 µm cutter diameter (TR-2-0100-S, PMT, Longmont, CO, USA) was mounted. This tip precisely carved a 75 mm (L), 25 mm (W), 1.5 mm (H) rectangular substrate of poly-methyl methacrylate (PMMA; transparent acrylic sheet, Newton Acrylics, Tlalnepantla, Mexico). After fabrication, the PMMA layer was completely perforated with the desired design and cleaned using compressed filtered air (3 bar), isopropyl alcohol, and, finally, deionized water.

In order to obtain a hermetically sealed microfluidic chip from the perforated PMMA layer, two lids of the same dimensions were necessary. For this laboratory prototype version, a glass slide (VE-P10, Velab, CDMX, Mexico) was used for the top lid, and a 380 µm thick slab of flat, pristine PMMA was cut with the same dimensions to serve as chip bottom (GF86267751, Sigma Aldrich, Steinheim, Germany). Note that a thinner piece was selected for this bottom lid to guarantee a better interaction between the magnets and the beads. The two PMMA pieces were then carefully assembled: the two sheets were exposed to UV inside a longwave UV crosslinker with a 254 nm lamp (CL-1000L Series UV Crosslinkers, Analytik Jena, Upland, CA, USA) with an energy of 999,900 microjoules per cm^2^ for five minutes. This step activated the surfaces, increasing their wettability for optimal adhesion and sealing [34]. After the activation of the surfaces, a solvent bonding sealing technique [35] was used to attach both PMMA slabs together: 40 µL of pure ethanol (Supelco, Darmstadt, Germany) were dispensed onto the two faces prior to placing the lid on top of the microstructured layer. The formed chip was then placed in a pneumatic heat press (Dabpress, dp80 Pneumatic Rosin Press Machine) applying 2 bars of pressure at 70 °C for 2 min. Finally, after the sealing was concluded, the chip was left to cool at room temperature for at least 5 min before further manipulation.

#### 2.5.2. Loading of the Microfluidic chip

To avoid undesired internal mixing between wells, the connecting microchannels were filled with 1.6 µL of mineral oil (M5904, Sigma-Aldrich, Steinheim, Germany) through low retention micropipette tips using the small filling ports located at the center of each channel (Figure S2). The filling ports were then dried using a lint-free wipe and sealed with a piece of adhesive tape to avoid any unwanted displacement of the buffer-oil interphase meniscus during the chip handling. The mineral oil used here served as a separating phase between wells, allowing only solid transport (beads) with the external magnets and motorized platform but retaining the liquid content of each well, avoiding unwanted species to pass through Supplementary Information S1.1 and videos SV1 to SV6. Once all the microchannels were filled with the oily phase, wells were filled with 10 µL of their corresponding solution using a different low retention micropipette tip. The wells were filled in decreasing order, having wells 6, 5, and 3 PBS-Tween 0.01%, well 4 αHIgG-488 solution, well 2 the patient serum solution (1:125 in PBS-Tween 0.01%), and well 1 PS-functionalized magnetic beads solution (80 K beads). It is important to note that Tween was used as a surfactant to reduce surface tension between beads and the surrounding media, and different percentages of dilution were tested, yielding 0.01% as the optimized concentration (Supplementary Information 1.1 and videos SV7 and SV8). Finally, after filling all the wells, a cleaned glass cover slide was placed on top of the microfluidic chip to seal it and prevent contamination of the wells, and the chip was placed on the automated platform.

#### 2.5.3. Control of Bead Displacement Inside the Microfluidic Chip

A custom-made automated platform was also constructed for a robust and more reproducible manipulation of the magnetic beads inside the microfluidic chip. The motorized setup (shown in Figure 1D and Supplementary Information Figure S1.2.1) allowed to precisely displace the magnetic beads inside the chip using a set of three cylindrical neodymium magnets (grade N50) of 3.5 mm (H) and 5 mm (D) each, arranged linearly with a center-to-center separation of 7 mm. First, the chip was fixed inside a rigid frame attached to a stepper motor for a precise, programmable longitudinal displacement, and the magnets were fixed above and below the plastic microfluidic chip with only a vertical degree of freedom, allowing them to approach or recede the chip and control the precise manipulation of beads thanks to magnetic attraction at a distance (380 μm) through the plastic walls. For the well-to-well displacement (Supplementary Information Figure S1.2.1A), first, the magnet below the chip was approached to gather and immobilize the beads at the bottom of the well, and the whole chip was displaced along its x-axis to transport the beads towards the following well at a controlled speed. After an optimization process to identify the best transfer speed, the beads were displaced at 10 mm/min inside the wells and intercalated microchannels. The speed was, however, set at 6 mm/min at the buffer/oil interface to avoid a potential loss of beads due to surface tension in case of rapid crossing. 

To increase reproducibility and favor the interactions of beads with the complete volume of the sample (well 2) and the anti-human IgG APAA-αHIgG-488 (well 4), a vertical stirring routine was programmed during the whole reaction time (Supplementary Information S1.2). For that purpose, an additional set of three magnets with the same degree of freedom as the other set of magnets (transversal to the longitudinal axis of the microchannels) was placed above the chip. This was indeed found to improve the homogeneity of the reaction.

Finally, at the location of the detection well (well 6), a simple combination of vertical and lateral movement was used to impose a flat spreading of the beads across the bottom surface of the well (Supplementary Information Figures S1.2.1C and S1.2.3). This coupled movement of both the magnets and the platform in a diagonal ramp indeed helped maximize the surface area occupied by the beads and avoid any shielding effects on the fluorescence intensity detection due to beads being agglomerated in a specific area of the well. The complete movement of the beads inside the chip can be observed in the Supplementary video SV9.

#### 2.5.4. Optical Detection

After the beads were spread in a controlled way at the bottom of the detection well, the chip was carefully removed from the automated platform and placed on an ad hoc holder for posterior image acquisition on an inverted fluorescence microscope (Cytation 5, BioTek, Winooski, VT, USA), focusing the beads inside the detection well using bright field microscopy. Once focused, three different regions of interest with low bead agglomeration were photographed using filters: GFP (EX 469/35, EM 525/39) and CY5 (EX 628/40, EM 685/40) with a 20X magnification, an exposure time of 164 milliseconds, and a gain of 15. The images acquired were analyzed using the procedure described in Supplementary Information S2. In the future, a dedicated optical sensor is developed and validated against this much more precise method (but still limited to a lab’s use).

### 2.6. Statistical Analysis

**Figure 2:**
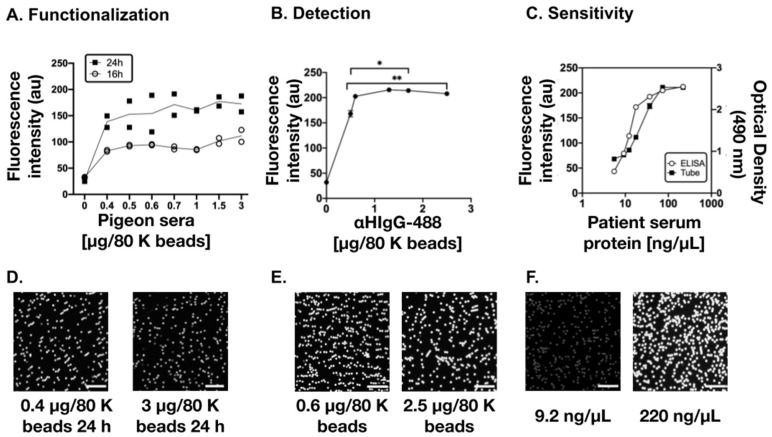
Optimization of the Diagnostic Biosensor Method. (**A**) Determination of pigeon serum (PS) concentration for functionalization and incubation time. Fluorescence intensity (au: arbitrary units) obtained with different concentrations of PS per sample (80 K beads) during 16 h (white dots) or 24 h (black squares) of functionalization incubation. (**B**) Determination of the optimal concentration of the secondary antibody (αHIgG-488) concentration, at which the fluorescence intensity saturates. Data are represented as mean ± SD of n = 4 experiment, *p* < 0.0001. (**C**) Comparison of the sensitivity range of the ELISA and magnetic beads methods. Data are represented as mean ± SD of n = 3 experiment, *p* < 0.0001. (**D**–**F**) Representative microscopy images of (**A**–**C**), respectively. Scales: 50 µm. Reaction platforms were 1.5mL low binding microtubes.

A student’s *t*-test was used to calculate statistical significance for Figure 2A, and a *p*-value < 0.05 was considered to be significant. A paired *t*-test was considered for Figure 2A, whereas an unpaired *t*-test was used for Figure 2B,C and Figure S4A. Kappa and ROC analyses were performed with IBM SPSS Statistics for Mac, version 26 (IBM Corp., Armonk, New York, NY, USA).

## 3. Results

### 3.1. Optimization of the Diagnostic Biosensor Method

To optimize the DBM before applying it directly on the microfluidic chip, different parameters of the three reaction phases described before were optimized. First, we determined the concentration of PS for functionalization and the incubation time that allowed the capture of the greatest amount of IgG APAA in the samples (method step 1). The fluorescence intensity obtained with functionalized beads with PS solutions at different concentrations (0, 0.4, 0.5, 0.6, 0.8, 1, 1.5, 3 μg/80 K beads) and incubation times (16 h vs. 24 h) were compared. 

In the DBM steps 2 and 3, serum antibodies from an HP-positive patient (18.3 μg/80 K beads) and secondary antibody αHIgG-488 (1.7 μg/80 K beads) were used; both reactions had incubation times of 2 h and with 1.5 mL low-binding microtubes as the reaction platform. The results depicted in Figure 2A show that 24 h incubation of the beads with PS 3 µg/80 K beads provided a greater fluorescence amplitude ranging from 54% to 93%.

Once the optimal functionalization conditions were defined (PS 3 μg/80 K beads, 24 h), the optimal αHgG-488 concentration required to obtain an acceptable level of detection was determined, referred to as saturation in fluorescence intensity, allowing to discern the range of IgG APAA in the patient serum. For this purpose, tests in microtubes were carried out in which the concentration of the HP-positive patient serum (18.3 μg/80 K beads; 2 h incubation) was maintained, but different concentrations of αHgG-488 (0, 0.5, 0.6, 1.3, 1.7, 2.5 μg/80 K beads; 2 h incubation) were evaluated. The results showed that the saturation in fluorescence intensity was reached at concentrations of 0.6 and 1.3 μg/80 K beads (Figure 2B).

Once DBM step 1 and 2 were settled, in order to determine the sensitivity, a dose-response curve using different concentrations of HP-positive patient serum (2.8, 4.6, 6.1, 8.8, 18.3, 36.7, 100 μg/80 K beads, 2h incubation) was performed using microtubes. To compare with the ELISA, the following equivalent concentrations were used, respectively: 5.5, 9.2, 12.2, 17.6, 36.7, 73.3, 220 ng/μL, in 500uL. Furthermore, the comparison was made with the usual employed ELISA using the same patient serum (Figure 2C). The sensitivity of both methods was compared and presented a similar tendency. In both methods, the two lowest concentration points were significantly different between them (*p* = 0.004 for DBM), and saturation of the signal was observed at around 73.3 ng/μL (no significant difference between the concentrations of 73.3 ng/μL and 220 ng/μL, corresponding to 36.7 and 110 μg/80 K beads, respectively). Representative microscopy images of the magnetic beads functionalization and reaction are also illustrated in Figure 2D–F. From these results, it could be seen that the dynamic range within the concentrations studied was the same for both methods.

To verify whether there was a non-specific binding of patient serum or αHIgG-488 to the non-functionalized and functionalized magnetic beads, controls with different conditions on the three steps of the DBM were performed in microtubes and compared to a full reaction that was PS-functionalized beads, patient sample and secondary antibody αHIgG-488 (Table 1 and Supplementary Information Figure S3.1). It could be seen that all the tested conditions gave a fluorescence intensity lower than that of the full reaction; in four of the five conditions, this signal was less than half of that of the full reaction.

According to the literature, the detection limit signal of an analytical method should be greater than three times the standard deviation of the blank measurement [36]: to take into account noise from non-specific binding, Control 5 was used to calculate a minimum signal for the limit of detection (LOD) of 58.3. This indicated that the LOD of this method could be lower than the smallest concentration measured in Figure 2C of 5.5 ng/µL with a signal of 68.1 ± 3.0. In comparison, for the ELISA the minimum calculated signal for the LOD should be 0.22, which was also lower than the smallest concentration measured of 5.5 ng/µL with a signal of 0.52.

### 3.2. Detection of Human IgG Anti-Pigeon Antigen Antibodies in Patient’s Sera

**Figure 3:**
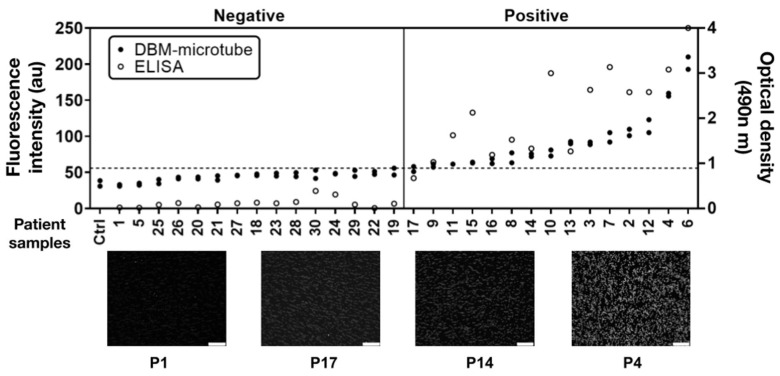
Comparison of the IgG anti-pigeon antigen antibodies detection in patient sera between ELISA and the Diagnostic Biosensor Method. A total of 30 patients’ serum samples were tested using the Diagnostic Biosensor Method (DBM), the fluorescence intensity (anti-human IgG Alexa 488) was measured by fluorescence microscopy (black dots ●), the absorbance of the same samples was measured by ELISA, right y-axis (white dots ○). The horizontal dashed line represents the microscope threshold for the reaction in the microtube and indicates the diagnosis of HP; higher values are considered positives and lower values negatives; the vertical line visually helps to differentiate negative from positive patients as diagnosed by ELISA. The negative control (Ctrl) corresponds to functionalized beads reacted with PBS instead of the patient sample and, subsequently, with αHgG-488. Samples were measured in duplicate. Representative microscopy images of patients 1, 17, 14, and 4 are shown. The white bar in the image corresponds to 75 microns. Reactions were performed in 1.5 mL low-binding microtubes.

To validate the DBM for HP, by detecting the presence of human IgG anti-pigeon antigen antibodies in sera, we tested 30 serum samples of HP and controls (positive and negative). The reactions of this test were carried out in microtubes: beads were functionalized with PS (3 μg/80 K beads; 24 h)—1.3 μg/80 K beads αHgG-488, and 0.08 μL patient serum/80 K beads were used—both reactions with 2 h of incubation time. As a negative control, the functionalized beads reacted with PBS instead of the patient’s serum and later with αHgG-488. The fluorescent intensity and the diagnostic obtained were compared against a conventional ELISA. The results shown in Figure 3 demonstrate that the DBM had a close correlation with the results obtained by the usual method of ELISA. 

A Receiver Operating Curve (ROC) was executed to assess the area under the curve (AUC) for the DBM as a diagnostic tool for HP. The AUC was very high (0.9667) and the cutoff point related to the highest specificity (96.67%), and sensitivity (100%) for the DBM variable was 55.80. The crosstabulation showed 96.67% sensitivity and 100% specificity, a positive predictive value of 96.67%, a negative predictive value of 100%. In order to determine the level of agreement between the ELISA and the DBM, we performed a Cohen’s kappa analysis. The level of agreement between the two diagnostic methods had a kappa value of 0.966 (*p* < 0.0001; Supplementary Information S4).

**Figure 4:**
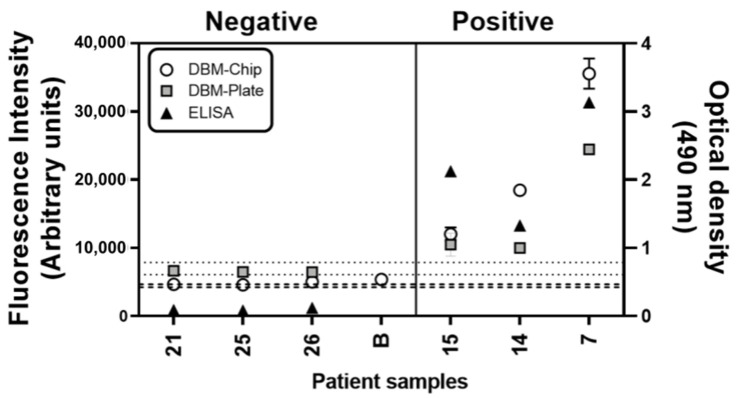
Detection of IgG anti-pigeon antigen antibodies in patients´ sera by Diagnostic Biosensor Method on microfluidic chip and plate. Six patient serum samples were selected (numbers correspond to sample numbers in our lab database), and IgG anti-pigeon antigen antibodies were detected (fluorescence intensity) by the Diagnostic Biosensor Method (DBM) on chip (white dots) and plate (gray squares). They are compared with ELISA optical density (OD) results (black triangles). Range detection limits (average +/− one standard deviation) are also indicated by the double line, threshold for the chip (---) and plate (⋯) were indicated. Data are represented as fluorescence intensity mean ± SD (B: whole blood patient sample).

Finally, the DBM was transferred to both a well assay plate and the microfluidic chip. As part of the developmental validation, six patient samples were selected from those previously evaluated in microtubes (three positives and three negatives) in order to compare between reaction platforms. The conditions of the reactions, for both well plate and chip, were 3 μg/80 K beads PS for 24 h for beads functionalization, 0.08 μL patient serum/80 K beads, and 0.63 μg/80 K beads αHgG-488. Incubation times for the patient sample and secondary antibody were changed from 2 h to 45 min (Supplementary Information S1.2.2). As seen in Figure 4, the diagnoses (positive or negative) were consistent between all used detection supports (well plate, microfluidic chip, microtubes, and ELISA). Interestingly, a whole blood patient serum was tested on the microfluidic chip and presented no noise signal (sample called B in the negative region).

The lower band of Figure 4 (bounded by the respective pairs of dotted and dashed lines) corresponds to the average +/− one standard deviation obtained from three repetitions of the negative control in each case (plate and microfluidic chip, respectively). It is seen from the thickness of these bands that the detection performed on-chip possessed a lower background noise (centered in 4455 with a σ = 412) than the detection on-plate (centered in 6990 with a σ = 1778). It can be noted, from Figure 3 and Figure 4, that the difference between negative and positive samples for the DBM was greater in the microfluidic chip, as the lowest positive sample signal to average negative sample signal ratio was 2.5 in contrast to that of the plate and tube that were 1.53 and 1.57, respectively. This result suggested that for the DBM, the microfluidic chip had a higher sensitivity than the microtubes or plate. It could also be seen that the ELISA had a higher positive to negative ratio of 5.7; this indicated that ELISA had a higher sensitivity. 

## 4. Discussion and Conclusions

The aim of this work was to develop a fast, cost-effective detection method for efficient diagnostics of HP looking for APAA in serum that allowed the future development of both a PoC and a high-throughput platform. 

In this context, we presented the DBM that, after standardization of the reaction steps, was validated in a plastic microfluidic chip, which can easily be scaled to high-volume manufacture in the future. The use of magnetic beads with an ad hoc motorized platform, made from readily available materials, allowed for a precise reaction and manipulation of the samples up to the optical detection stage; since an integrated standalone detector will be developed in the future, a commercial system was used.

Commercial magnetic beads ELISA was developed as Luminex^®^ xMap^®^, LEGENDplex^TM^, and FIREplex^TM^; while these methods represented important advances in the detection of multiple analytes for a single sample, they were not readily available. One of the main issues was that they required sophisticated and expensive equipment to obtain the fluorescence readings. The use of this equipment was not easy, and only trained personnel could do so; thus, they were made for large-scale use, limiting their potential use for a PoC device. Other initiatives of the use of magnetic beads on 96 well plates have been published [37]. In these methods, magnetic beads were used to support the reaction molecules and let the liquids pass through and not as reaction surfaces and carriers.

The use of microfluidic devices without the use of magnetic beads has recently been published [38]. These microfluidic devices represented a miniaturization of the ELISA method which, although they represented advantages (low sample/reagent consumption, multiple analyte detection, and high-throughput), their handling by the final user was not easy since they required multiple microfluidic connections, the replacement of liquids, and the acquisition and interpretation of images with specialized equipment.

Therefore, the work carried out here represented the conjunction of the two advantages published so far: the use of magnetic beads as a reaction and transport surface using the base of a competition ELISA and its adaptation to a microfluidic device with a motorized platform that automated the reaction process. 

Sensitivity and efficiency. Although the positive to negative ratio of the DBM was lower than that of the traditional ELISA, indicating lower sensitivity, the results obtained with the DBM, as demonstrated by ROC and kappa analysis, were in accordance with the measurements made with the ELISA employed on HP diagnosis. As shown in the results, the background noise ratio in this ELISA test was lower than that obtained for DBM. However, it was worth saying that the horseradish peroxidase (HRP) produced a colored, fluorimetric derivative when incubated with a suitable substrate. As its name indicated, it is an enzyme that performs two redox reactions, meaning that the background noise depended on time, temperature, and the handling of the inhibition reaction, so it represented a variable value. The DBM method of detection was fluorescent and, therefore, the noise value did not depend on the handling and was very stable from one test to another.

Patients with different anti-pigeon antigen antibody concentrations could be successfully detected and diagnosed. As demonstrated, the DBM could also be adapted for its use in microtubes, well plates, as well as microfluidic chips. One of the main problems of human IgG measurement using ELISA was that whole blood was not employed, and the serum had to be separated and diluted in order to obtain a correct signal. This complicated the diagnostic process and made it useless for the development of a PoC. Our results showed that in the microfluidic chip, the detection reaction was more efficient than the same reaction in plate and microtube, and the results from tested sera with varied antibody concentrations indicated a greater sensitivity. Moreover, fingerstick whole blood samples did not generate any noise signal (Figure 4); it was a promising sign indicating that it could be used directly using a simpler and less invasive method that would even reduce the costs. This validated the potential for future use in a PoC without the need for a previous phase of separation of the serum from the sample and dilutions to avoid background noise. Moreover, this DBM could be applied to less “invasive” samples as saliva.

Fast and automated detection method, the detection procedure of DBM lasted 1h 30min in a 96 well plate and 1h 10min in the microfluidic device in comparison with the minimum 3 h required for ELISA. The development of the custom-made automated platform for the controlled manipulation of the magnetic beads inside the microfluidic chip allowed the automation of the diagnostics. Therefore, the user would only have to put the sample in the chip well, and the platform would perform the displacement of the magnetic beads until the detection device might be employed, either individually or on a large scale with many samples at the same time, as in clinics and hospitals. In this case, it would limit the constant intervention of staff required in other methods. 

Remarks on the cost. The costs of performing DBM in American dollars were, for one sample in microtube 4USD, 96 wells plate 2USD, microfluidic chip 3USD, compared to the ELISA´s 6USD cost. In addition, as mentioned above, one of the great advantages of using the DBM on the microfluidic chip was that the user only had to put the sample on the chip well, and the platform automated the reaction process until the detection device might be employed. This represented a reduction in the costs associated with human error due to manipulation, as well as freeing up the user’s time for other matters.

Another big difference that influenced the cost was the use of laboratory equipment and material: in this case, the DBM was compatible with different reaction devices; therefore, its results could be read in multiple devices, from an ELISA plate reader to a microscope or a cytometer, among others. 

Point-of-Care and high-throughput diagnostic, the use of fluorescence as a marking signal was a wide field for which DBM could be used in multiple detections with the same sample, minor changes to the microfluidic chip could be carried out in order to achieve this goal. The chip design might be used for further reading using a fluorescence sensor for on-chip quantification and rapid diagnostics on a PoC implementation. This then needs to be implemented in the future and was outside the scope of the present phase of the project. However, an imaging system may already be employed for diagnostics; an automated program was also developed to be used from micrographs.

## 5. Conclusions

The DBM designed to detect the presence of human IgG antibodies in patient’s serum samples of avian-antigen-induced HP (tested in microtubes, well plates, and microfluidic chip) proved to be comparable for diagnostic purposes to conventional ELISA assays. The DBM principle was not limited to be used in an exclusive device of reaction. In conjunction with the microfluidic chip and a custom motorized actuated platform, it led to a number of advantages in the performance of the tests such as automation, reduced cost and testing time, the possibility of performing isolated or multiple samples, and no noise signal on a fingerstick whole blood sample. All these advantages, in addition to the use of fluorescent markers, led us to believe in a wide range of applications in biomolecules and equipment detection. We showed the signs of the development of a PoC, whose implementation could be possible in the near future, for biological and clinical applications other than avian-related hypersensitivity pneumonitis. This made it possible to offer a greater availability of analytic tests in places where the use of expensive and complex instruments is not an option.

## Figures and Tables

**Table 1:** Non-specific binding controls.

Condition	Reactions of the DBM			Fluorescence Intensity (au)	SD
	Step 1	Step 2	Step 3		
Negative control	PS	PBS	αHIgG-488	34.7	5.3
Control 1	PS	PBS	PBS	39.0	2.2
Control 2	Buffer 1	PBS	PBS	40.5	1.5
Control 3	Buffer 1	Patient sample	PBS	41.6	2.4
Control 4	Buffer 1	PBS	αHIgG-488	43.9	2.5
Control 5	Buffer 1	Patient sample	αHIgG-488	53.8	1.5
Full reaction	PS	Patient sample	αHIgG-488	101.6	4.3

DBM—Diagnostic Biosensor Method. Step 1—Beads functionalization (BSA blocked). Step 2—Reaction with IgG anti-pigeon antigen antibodies (APAA). Step 3.—Reaction with anti-human IgG APAA (αHIgG-488). PS—Pigeon serum. Patient sample used was patient namely three.

## Data Availability

All data relevant to the study are included in the article or uploaded as Supplementary Information. It’s available based on reasonable requests to the authors.

## Supplementary Materials

The following are available online at https://www.mdpi.com/article/10.3390/bios11060196/s1, Figure S1.1: Diagram of the final chip design, Figure S1.2.1: Principle of operation of the manipulation of beads for optimized stirring and reading, Figure S1.2.2: Improvement of the fluorescence signal using Transversal Magnet Movement (TMM), Figure S1.2.3: Results of beads spreading for three different platform / magnet combinations, Figure S2: Beads detection and analysis procedure, Figure S3.1: Flow cytometry results of non-specific controls test with DBM in microtubes, Figure S3.2: Flow cytometry results of patients test with DBM in microtubes, Figure S4.2: Receiver operating characteristic curve for Diagnostic Biosensor Method of Avian-related Hypersensitivity Pneumonitis, Table S4.1.1: Raw data used for comparative statistical analysis: DBM-microtube vs ELISA, Table S4.1.2: Diagnostic validity crosstabulation DBM vs ELISA, Table S4.1.3: Inter-rater reliability (Cohen’s Kappa, Table S4.2.1: Area Under the Curve, Table S4.2.2: Sensitivity and specificity.
