# Supplementary material for: Development of a Diagnostic Biosensor Method of Hypersensitivity Pneumonitis towards a Point-of-Care Biosensor
Source: Biosensors (Basel). 2021 Jun 15;11(6):196. doi: 10.3390/bios11060196 (PMC8232117; doi:10.3390/bios11060196)
Supplement: Supplementary file 1 [file biosensors-11-00196-s001.zip › biosensors-1182377-supplementary.pdf]

40Supplementary Information

S1 Optimization of the chip design

S1.1 Avoiding mixing between neighboring wells

a. Selection of the best oil for separation

Several oily substances were tested to fill each microchannel that acted as a separating phase from well to well. This separation was needed to avoid mixed contents between adjacent wells while allowing the beads to be dragged through it by the magnet movement. The final selection of the oily phase used in the results reported above was based on avoiding the transmission of fluorescence through this phase while allowing fast filling of the microchannels (i.e. high front filling velocity). As it is seen when comparing the microchannel filling and fluorescence supplementary videos: SV1 (PBS filling), SV2 (Glicerol filling), and SV3 (Immersion oil filling) SV4 (PBS fluorescence), SV5 (Glycerol fluorescence), SV6 (Immersion oil fluorescence) both the mineral oil and the immersion oil accomplish the properties mentioned above but we opted for the former because of its cost effective price.

b. Implementation of intermediate wells

During the preliminary steps of the project, experiments were performed to evaluate the possible hauling of unbound species from one well to another only by passive dragging during beads movement using the magnets. The results persistently showed that a very small, yet slightly detectable, amount of unwanted antibodies was passively transported by the controlled movement of the beads from the reaction wells to the following well, thus disrupting the further optical readings (depicted schematically in Figure S1.1B for wells 3 and 5). However, it was possible to cancel this undesired hauling effect by adding an intermediate well with only buffer; this additional step thus acts as a quick washing step to dilute the unwanted unbound species that are not supposed to be counted in the final reading by adding a buffer well and 2 extra

separation menisci blocking passive transport. This is the explanation for the 2 extra wells (wells 3 and 5) in the final design.

c. Selection of surfactant

Tween was incorporated to the buffer 4 solution to act as a surfactant, increasing the wettability of the well surface and avoiding the beads to get stuck in the irregularities of the same when they are being dragged by the magnets. However a high tween concentration is not advisable because any small amount of buffer content hauled by the beads to the oily phase during movement will act as an anchor to the surface of the chip and the beads will thus get stuck to the surface in small traces. Furthermore, it was not known if high concentrations of tween would inhibit the reactions of beads with the antibodies present in the serum patient sample (which contains IgG anti-pigeon antigen antibodies) and also with the secondary antibodies for fluorescence reading (αHIgG-488).

After evaluation of the loss bead content and the effect tween has on the final fluorescence reading (not reported) for two different concentrations diluted in buffer 4 (see Supplementary videos SV7 (Tween 0.01%), and SV8 (Tween 0.1%)) the 0.01% tween concentration was selected as the optimal concentration.

In the supplementary videos it is seen that at high tween concentration (0.1%) the beads velocity inside the well when the magnets are brought close to the surface is notably faster than at the low tween concentration (0.01%). This is due to the fact that wettability of the surface is higher when Tween concentration is higher and hence the beads are more loose to move.

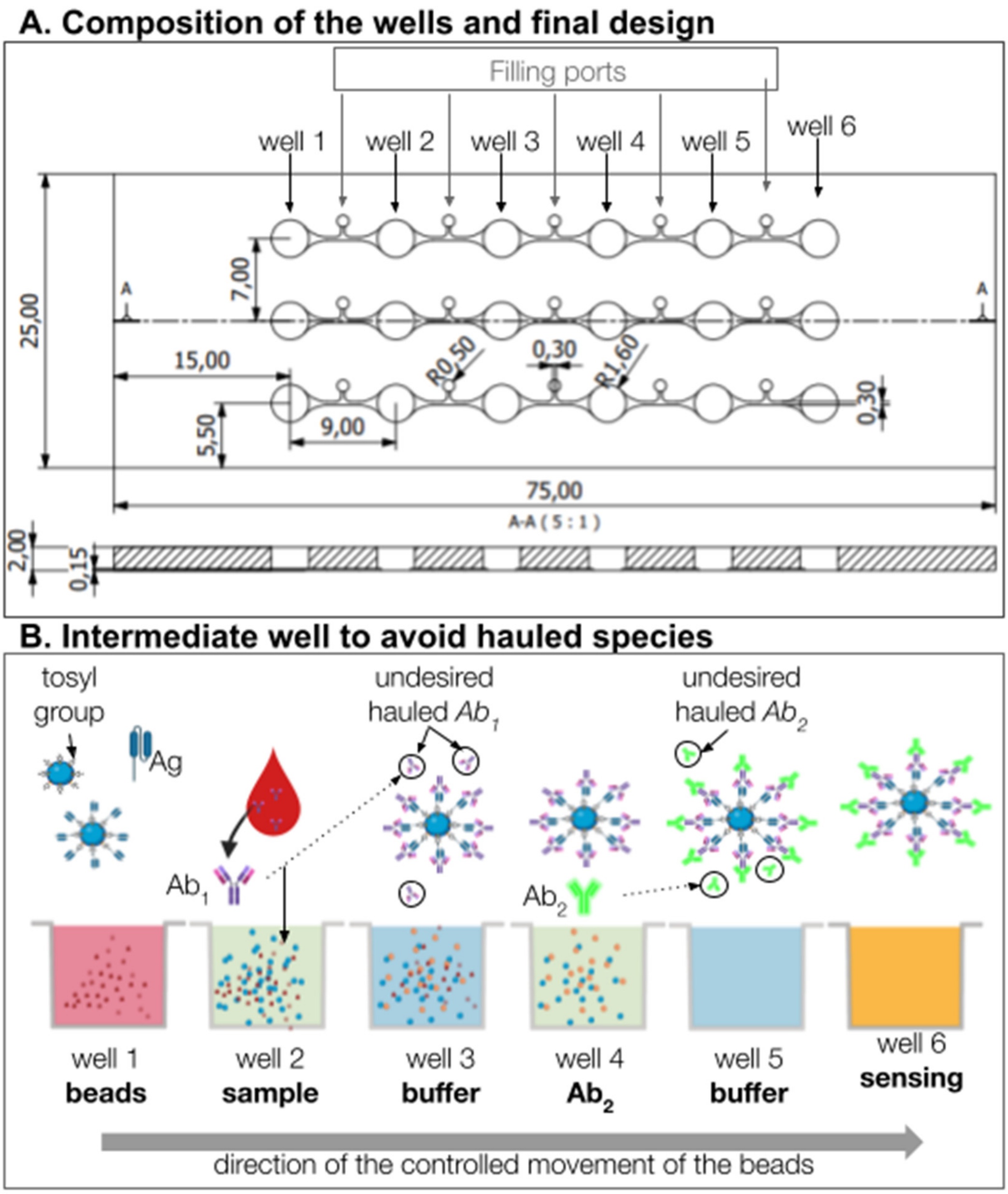

Figure S1.1. Diagram of the final chip design.
A. Dimensions of the final functional design (in millimeters). Small filling ports were used to charge the microfluidic channels with the oily phase to avoid undesired internal mixing between wells.
B. Details of chip content is shown to illustrate that some unwanted species may be transported passively by following the movement of the beads while they are displaced using the magnets. These small amounts of hauled antibodies found in the wells immediately following the reaction wells were cancelled out by adding intermediate wells (wells with numbers 3 and 5) filled with buffer.

S1.2 Automated magnet platform for beads manipulation on chip

a. Transverse magnet movement

To optimize both the reactions of the beads with the antibodies present in the patient sample (containing IgG anti-pigeon antigen antibodies) and also with the secondary antibodies for

fluorescence reading (αHIgG-488), two different routines were implemented on the chip platform. First, a vertical stirring routine where the magnets are displaced transversally to the microchannel longitudinal axis and a spreading routine where the magnets and the chip are displaced in a coupled movement once the beads are inside the detection well, prior to the fluorescence intensity detection. During the reaction of beads with the patient sample containing IgG anti-pigeon antigen antibodies and also with αHIgG-488, agitation of beads was accomplished by movement of both the immobilization magnet and the mixing magnet. The magnets were moved simultaneously in the vertical direction, transversal to the microchannel longitudinal axis, as seen in panel B of Figure S1.2.1. In step (3) the mixing magnet is brought into close contact with the upper surface of the chip, while the immobilization magnet moves away from the lower surface of the chip, this creates an uplift effect on the beads, the magnets remained fixed in this configuration for 2.4 s and afterwards, in step (4), the magnets were moved in the opposite direction and the beads were dragged down again, magnets also stayed in this configuration for 2.4 s. Steps (3) and (4) were repeated during the whole reaction time. This movement can be observed in Supplementary Video SV9.

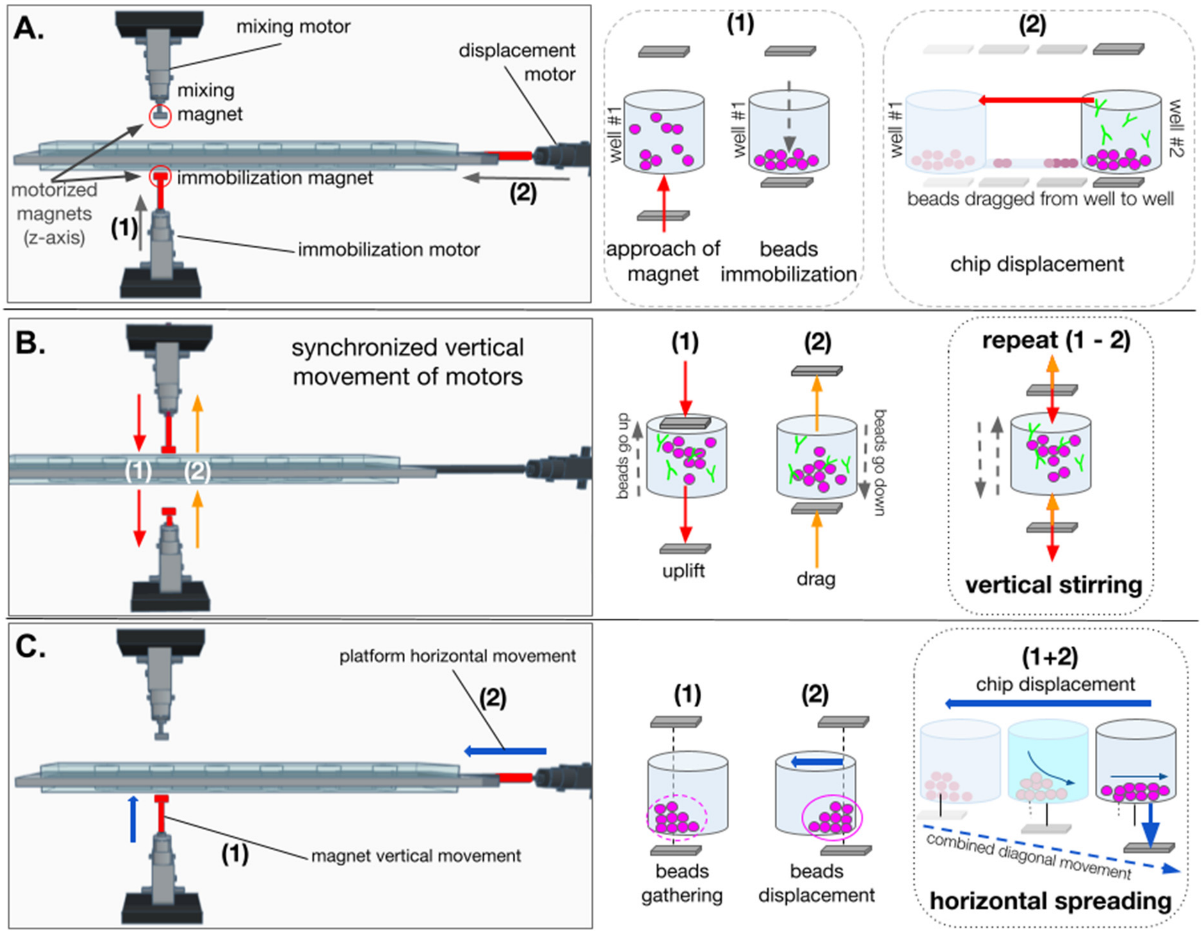

Figure S1.2.1. Principle of operation of the manipulation of beads for optimized stirring and reading.
A. Principle of operation of beads displacement. The magnet below the chip was approached to gather and immobilize the beads at the bottom of the well (1) and the whole chip was displaced along its x-axis (2) to transport the beads towards the following well.
B. Transversal Magnet Movement (TMM) is a vertical stirring used when beads are inside the wells in which they react with primary and secondary antibodies. The two magnets that are coupled to a vertical movement are displaced in a synchronized fashion, in order to slowly attract the magnetic beads up (1) and down (2) consecutively inside the well, optimizing the contact with the antibodies that are dispersed inside the well. This vertical stirring is performed at a fixed velocity of … during the whole reaction process.
C. Horizontal spreading of the beads at the well floor. By correctly programming the combination of a vertical displacement of the chip (1) with a vertical movement of the magnets (A-1 and A-2), it is possible to control the correct horizontal spreading of the beads on the floor of the desired well.
The complete operation of the manipulation of beads can be observed in Supplementary Video SV9.

To study the effect of Transversal Magnet Movement (TMM) and to set a correct reaction time for the beads to bind with the IgG APAA antibody contained in the patient sample (well 2) and with the secondary antibody αHIgG-488 (well 4), the functionalized beads were set to react with three increasing concentrations of IgG APAA from patient serum at three different reaction times (15, 45 and 120 min), with and without TMM. The results are shown in Figure S1.2.2

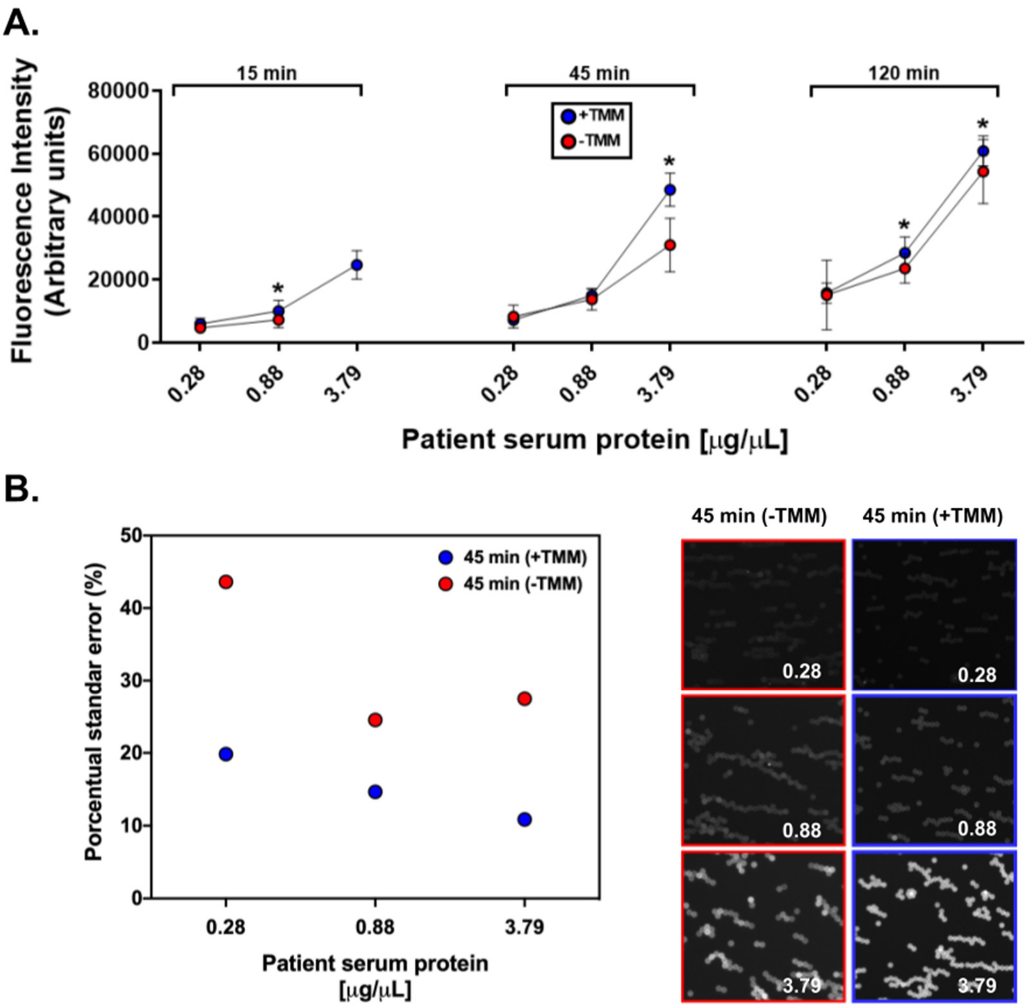

Figure S1.2.2. Improvement of the fluorescence signal using Transversal Magnet Movement (TMM). A. Comparison of sensitivity curves at three different reaction times (15, 45 and 120 min) with (+TMM) and without (-TMM) Transversal Magnet Movement, where the error bars represent the SD of the average value over three repetitions. Asterisks denote significant differences ($P<0.05$)
B. Influence of TMM on the quality of the captation of αHIgG-488 by the beads. Left panel is a comparison of the width of the Gaussian distribution of fluorescence intensity of beads with TMM (blue) and without it (red). Right panel shows micrographs of selected areas with fluorescent beads for each condition.

As it is seen in panel A, the fluorescence reading of the increasing IgG APAA concentrations increases in all reaction times when TMM is done, this reflects the fact that captation of human IgG anti-pigeon antigen antibodies in patient sera and αHIgG-488 antibodies is greater when beads are being mixed during the reaction.

Furthermore, in panel B the standard deviation of the gaussian distribution fitted to the fluorescence intensity readings with the python analysis program was plotted for the 45 min reaction time sensibility curve. It is evident that when TMM is present the width of the gaussian distribution is smaller than when TMM is not present. For example, in the high end

concentration of the sensibility curve (3.79 μg/μL of patient serum) the width of the gaussian distribution is approximately 10% its median with TMM, while the same parameter is approximately 30% without TMM. This can be seen in the micrographs of panel B, where the brightness of beads is more stable with TMM than without it.

The detected fluorescence is highest at 45 min in comparison to the 15 min , even if at 120 min we observed the greatest detection, we notice that part of the contents on the wells evaporated, so we discarded this reaction time. After these experiments, 45 min reaction time with TMM was selected as the optimal reaction routine, and was used in the experiment reported in this paper.

b. Optimization of the spreading of beads inside the chip

To reduce possible fluorescence screening effects by beads being agglomerated during the detection, three basic platform/magnet movement routines were investigated to maximize the final area occupied by the beads in the detection well. The first routine was an horizontal continuous displacement of the platform while leaving both the mixing and immobilization magnets fixed with the later one being immediately below the surface of the chip, this is seen in the right side of panel A in Figure S1.2.3. Secondly a coupled movement of the platform in the horizontal direction and of both magnets in the vertical direction was used to create an overall diagonal movement of the beads inside the well, this is shown in panel B. Finally, depicted in panel C, the same diagonal movement as in B was complemented with a final reverse movement of the platform to be able to spread the beads over a broader area with the influence of the mixing magnet being closer to the surface of the chip and creating an overall “C” shape movement of the beads.

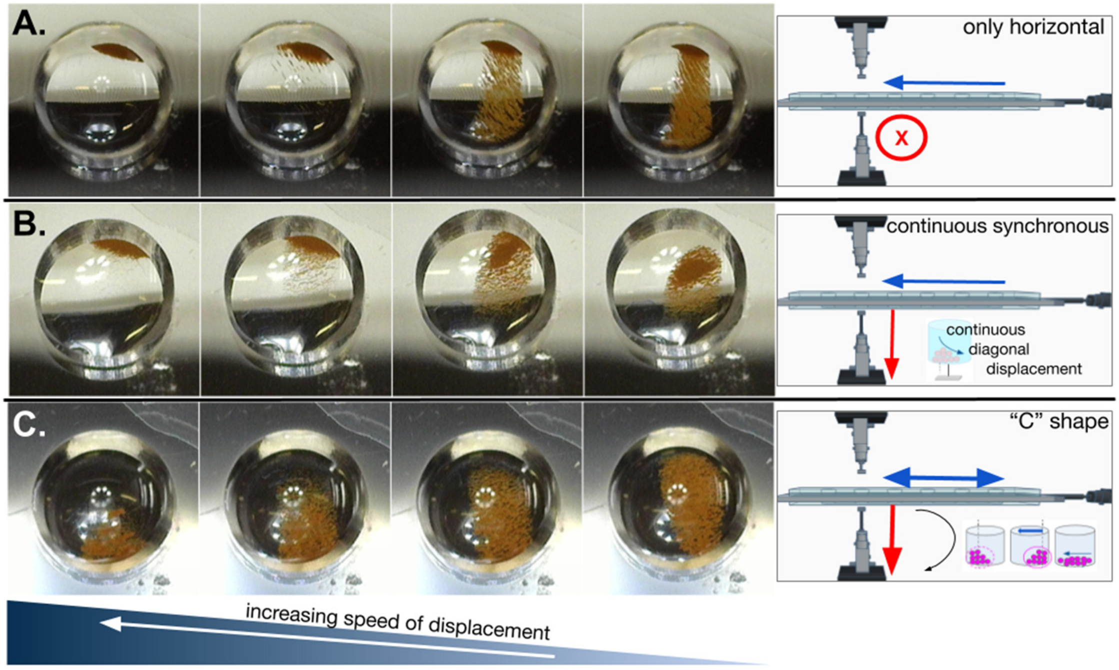

Figure S1.2.3. Results of beads spreading for three different platform / magnet combinations.
Comparison of three different platform/magnet movements for spreading the beads inside the detection well. An increasing velocity gradient was set and the configuration which maximized the area occupied by the beads smudge was finally selected as the optimal one.
A. Horizontal displacement of platform with vertical magnets being fixed in one position, where the immobilization magnets is immediately below the surface of the chip.
B. Horizontal platform displacement coupled with vertical displacement of magnets which creates an overall diagonal movement of the beads smudge.
C. Same as in B with an additional reverse horizontal movement to spread the final beads smudge over a broader area of the well

After implementing a velocity gradient of platform/magnets in each of the three movement routines, we selected the "C" shape because it maximized the final area occupied by the beads inside the detection well, as can be seen in the comparison of the final configuration of beads after applying each movement routine with the velocity gradient in the left side of Figure S1.2.3. This movement can be observed in Supplementary Video SV9.

S2 Beads detection and analysis

The obtained images were analyzed using a Google Colaboratory notebook with Python 3. Beads in the images were detected using a Laplacian of Gaussian filter (LoG) through the function *blob_log()* function from the scikit-image library [1]. This function performs a blob detection in grayscale images having bright blobs in a dark background, see Figure S2A, by

means of a scale-space representation. The idea behind this procedure is to generate a set of 2D images, using as a basis the original image, every image in the set is created applying a LoG filter to the original image using an increasing value of the variance σ^2 of the Gaussian kernel, the value $t = \sigma^2$ is denoted as scale parameter [2]. The increasing variations of the scale parameter allow it to suppress the fine details in the image, highlighting the coarser objects in the resultant image after the Laplacian operator. Finally, the set of images is arranged in a volume and the points that are local maximum on both parameters, scale and space, indicate the possible presence of a blob, being the scale at which they are maximum proportional to the square of the radius while its brightness indicates the relevance of the blob [3].

Regarding the running of the *blob_log* function, it has 7 different parameters that may be varied in order to adequately find the blobs in images, where three of them are the most relevant for the purposes of the beads detection procedure used here: *min_sigma*, *max_sigma* and *threshold*. The first two parameters allow establishing an interval of the radius size for the searched blobs, since the radius r, in pixels, is related to the scale parameter t through $r = \sqrt{2t}$ [1], *min_sigma* and *max_sigma* parameters were determined by measuring manually the approximate diameter of the beads using ImageJ software and computing $\sigma = r/\sqrt{2}$. The diameter was measured here because it was easier to draw than the radius line and induced less error. Based on the computed σ value, min_sigma and max_sigma were determined having values close to σ (because the size of the beads in images was almost constant). When these two sigma parameters were defined, they were fixed for all the images acquired with the same specifications. The third critical parameter is the threshold; it specifies the lower limit from which local maxima with a given low intensity brightness needed to be rejected in the final detection. Since the intensity of beads presented variations among different conditions, this parameter was modified manually from image to image, but remained unchanged for images originating from the same sample. Here, the threshold parameter values varied from 0.001 to 0.05. The other four parameters were set to their default values as stated by the

function in the scikit-image library and for all the images, with the exception of the *num_sigma* parameter which was set to 1 (this parameter indicates the number of intermediate values between min_sigma and max_sigma that are going to be considered for blob detection, i.e. the number of scale parameters that are going to be computed, which could indeed be helpful to detect beads of different sizes, but here the beads radius was was considered to be nearly constant). Finally, the blob_log function returns a list of 2D coordinates indicating the center of each identified bead together with its associated σ value which allowed computing the radius of every bead in pixels, using $r = \sigma\sqrt{2}$.

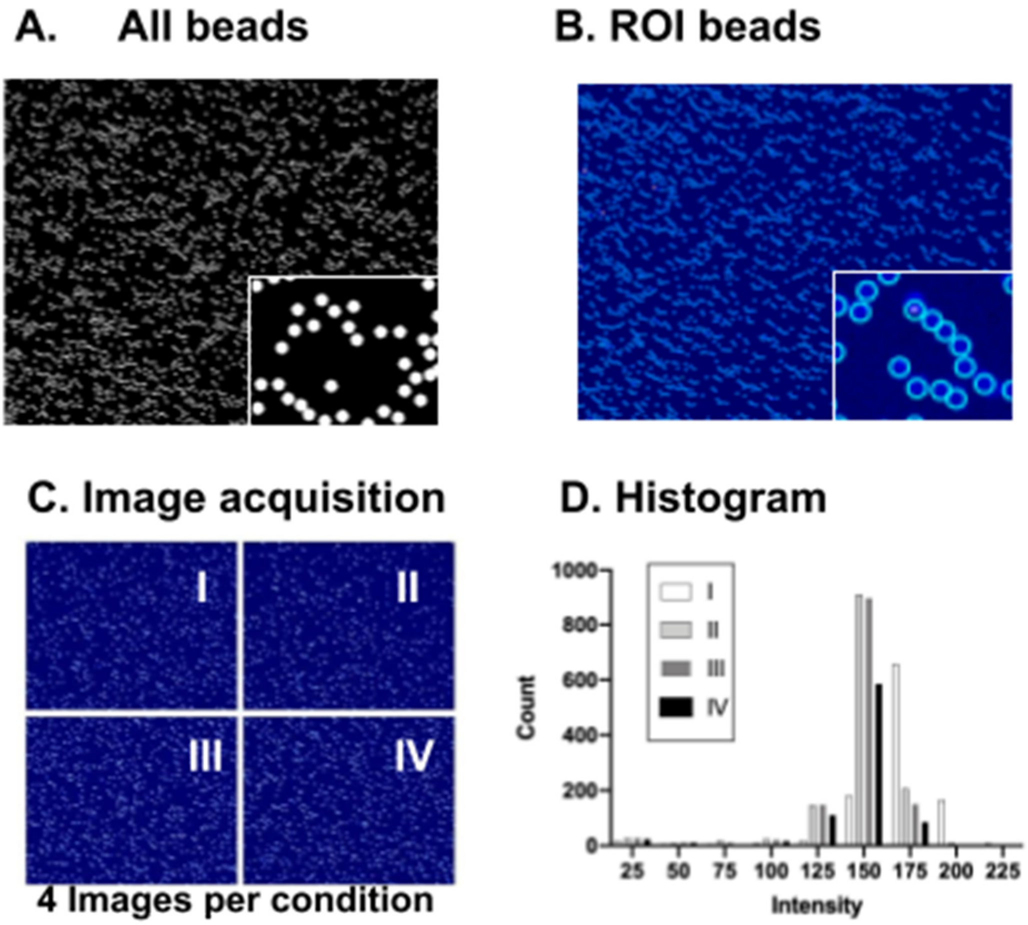

Figure S2. Beads detection and analysis procedure.
A. Image showing the beads as bright blobs in a dark background. This image is the input for the blob_log() function from the scikit-image library.
B. Example of the output images of the program, showing the original image A in pseudocolor with the detected beads inside the circular ROIs in cyan, it helps to perform a fast checkup of the performance of the program.
C. Example of the resultant output ROI images for 4 different input images with the same condition, the information obtained from these detections is used for the creation of the histogram shown in D.
D. Complete histogram computed using the information of the individual images under the same condition in C, individual information is denoted by different shades of the grayscale. The information of mean, standard deviation,

mode, etc., is obtained from this complete histogram and is used to plot the mean fluorescence intensity for each condition analyzed.

Thanks to the collection of the information of the position and radius of the beads located in the image, two images associated with the original image are generated (Figure S2A). The first one is a binary image that indicates clearly the area occupied by each detected bead in the original image with white pixels. The second one is the original image in pseudocolor with the regions of interest (ROIs) highlighted in cyan, Figure S2B. The binary image helps to overlay the ROIs on the original image using ImageJ software for a careful inspection of the detection performance, while the pseudocolor image allows a rapid manual verification. Because this was implemented for every acquired image, the final result consists of a pseudocolor image per photograph of four images of any given condition, Figure S2C.

Regarding the performance of the detection of the beads, it was observed that the LoG filter had difficulties to clearly discriminate between beads when located inside agglomerates. Sometimes, it could return boundaries that were slightly displaced relative to the beads center. This occurred because the fluorescence profile of the beads did not fit perfectly a smooth function like a Gaussian. Variations were typically found across the bead area and these variations forced the boundary to move around the maximum of fluorescence inside the bead, which sometimes did not correspond to the bead center.

In addition to the aforementioned outputs, information about the fluorescence intensity was collected in an image from each identified bead; it includes the mean, maximum value, minimum value and standard deviation. Using this piece of information, an histogram of beads fluorescence intensity was constructed and plotted together with the mean, standard deviation, mode and the width of the histogram in one image, Figure S2D. The width of the histogram was computed through the second quartile of the intensities converted as a percentage of the mode. Then, a computation of the mean, standard deviation, mode and the width of the histogram was performed for each condition, taking into account all the images belonging to the given condition. This calculation was finally used to plot the variations of the average

fluorescence intensity for all the different conditions in a single graph. All the information related to both the individual images and the collection of images for a given condition was finally saved in a spreadsheet-compatible file. For the computation of the fluorescence intensity, all the pixels inside a ROI were taken into account.

References

1. Van der Walt, S.; Schönberger, J.L.; Nunez-Iglesias, J.; Boulogne, F.; Warner, J.D.; Yager, N.; Gouillart, E.; Yu, T. Scikit-Image: Image Processing in Python. PeerJ **2014**, 2, e453.
2. Wah, B.W. Scale-Space. In Wiley Encyclopedia of Computer Science and Engineering; John Wiley & Sons, Inc.: Hoboken, NJ, USA, **2007**; p. 1019.
3. Lindeberg, T. Feature Detection with Automatic Scale Selection. Int. J. Comput. Vis. **1998**, 30, 79–116, doi:10.1023/a:1008045108935.

S3 Control measures by Flow cytometry

To evaluate that the reaction IgG APAA- anti human IgG APPA (αHIgG-488) occurred only in the presence of the antigen of interest, we tested several conditions to check that the controls were appropriate and there was not non-specific fluorescence. These conditions were performed with DBM in microtubes and read with fluorescence microscopy (Table 1) and Flow cytometry. The latter was performed using a Beckman Coulter Cytoflex cytometer. In the conditions previously described a total of 5,000 events from the αHIgG-488 gate were acquired. The analysis was done using a FlowJo V10 Software (Beckton Dickinson, Ashland, Or, USA) and shown in Figure S3.1.

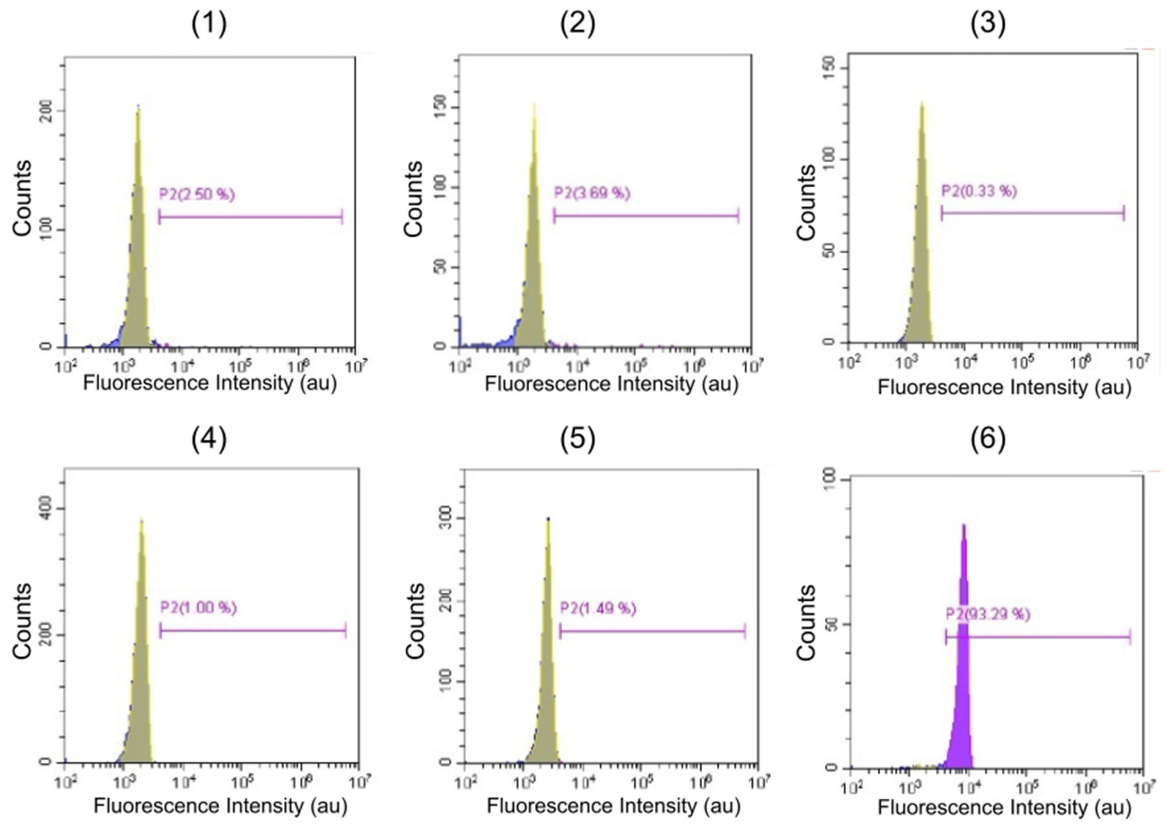

Figure S3.1. Flow cytometry results of non-specific controls test with DBM in microtubes.
(1) PS-functionalized beads. (2) Non-functionalized beads. (3) Patient sample on non-functionalized beads. (4) αHIgG-488 on non-functionalized beads. (5) Patient sample and αHIgG-488 on non-functionalized beads. (6) Full reaction. Patient sample and αHIgG-488 on PS-functionalized beads.

We also evaluated a negative control (PBS instead of patient sample) and patients of known diagnosis (three positives and one negative). It can be seen in Figure S3.2 that the reaction was able to resolve the positive patients from the negative patient and the control.

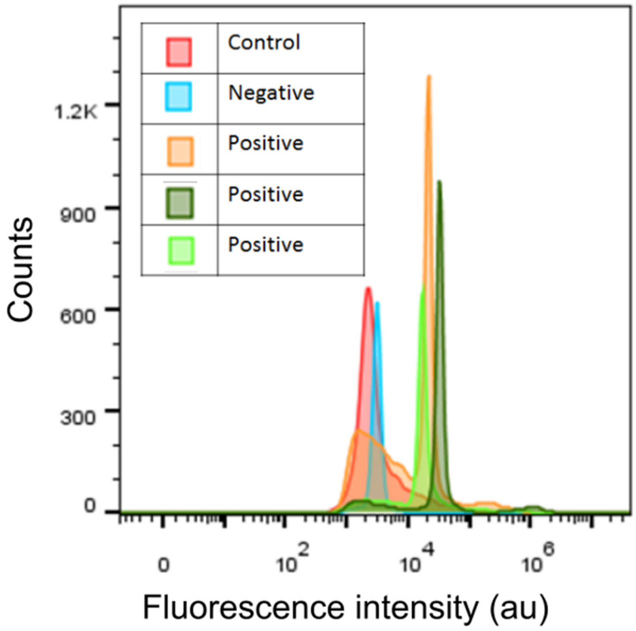

Figure S3.2. Flow cytometry results of patients test with DBM in microtubes.

S4 Comparative statistical analysis between the Elisa technique and the Diagnostic Biosensor Method

S4.1 Crosstabulation

Table S4.1.1 Raw data used for comparative statistical analysis: DBM-microtube vs ELISA

Patient sample number	Fluorescence intensity (au) for the DBM-microtube		ELISA (optical density 490nm)	SD
	Repetition 1	Repetition 2		
Ctrl	30.9	38.5	-	-
1	30.9	33.0	0.021	0.007
5	32.3	34.9	0.017	0.005
25	34.2	40.2	0.083	0.007
26	41.0	43.3	0.121	0.010
20	40.9	43.5	0.025	0.008
21	39.2	45.3	0.088	0.015
27	45.3	46.1	0.117	0.007
18	45.5	47.9	0.129	0.002
23	44.8	49.0	0.116	0.006
28	44.1	49.7	0.146	0.009
30	41.6	53.0	0.388	0.007
24	47.7	48.6	0.309	0.004
29	44.3	52.9	0.087	0.003
22	47.2	51.3	0.010	0.004
19	46.4	55.8	0.108	0.018

17	51.0	58.1	0.672	0.004
9	57.2	61.5	1.028	0.053
11	61.5	62.1	1.623	0.036
15	63.0	64.1	2.124	0.014
16	62.2	68.7	1.189	0.009
8	63.3	77.1	1.523	0.015
14	71.8	75.7	1.328	0.021
10	72.6	81.0	2.998	0.021
13	89.7	92.7	1.267	0.145
3	88.6	92.0	2.630	0.087
7	92.0	105.1	3.135	0.064
2	100.8	109.7	2.577	0.040
12	105.1	123.0	2.581	0.035
4	155.6	159.5	3.080	0.057
6	192.7	210.0	>4.000	-

Table S4.1.2 Diagnostic validity crosstabulation DBM vs ELISA

			ELISA		
			Negatives	Positives	Total
DBM	Negative	Count	30[b]	1	31
		% within ELISA	100.0%	3.33%	50.0%
	Positive	Count	0	29[a]	29
		% within ELISA	0.0%	96.67%	50.0%
Total		Count	30	30	60
		% within ELISA	100.0%	100.0%	100.0%

[a]True positives
[b] True negatives

Table S4.1.3 Inter-rater reliability (Cohen's Kappa)

		Value	Asymptotic Standard Error[a]	Approximate T[b]	Approximate Significance
Measure of Agreement	Kappa	0.966	0.033	5.552	0.000
N of Valid Cases		60			

a. Not assuming the null hypothesis.

b. Using the asymptotic standard error assuming the null hypothesis.

S4.2 ROC curve

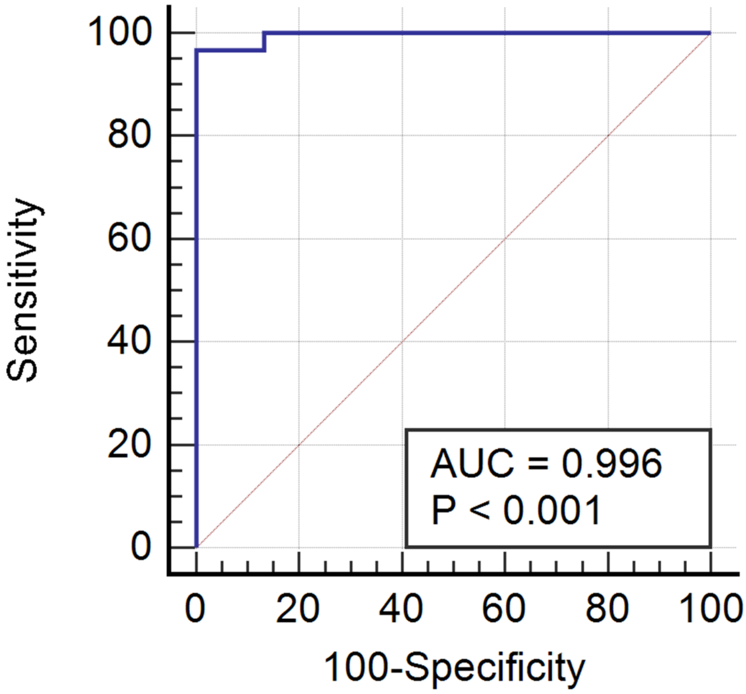

Figure S4.2 Receiver operating characteristic curve for Diagnostic Biosensor Method of Avian-related Hypersensitivity Pneumonitis.

Table S4.2.1 Area Under the Curve.

Test Result Variable(s): DBM

			Asymptotic 95% Confidence Interval	
Area	Std. Error[a]	Asymptotic Sig.[b]	Lower Bound	Upper Bound
0.996	0.005	0.000	0.932	1.000

Table S4.2.2 Sensitivity and specificity

Criterion	Sensitivity	95% CI	Specificity	95% CI	+LR	-LR
≥ 30.921	100.00	88.4 - 100.0	0.00	0.0- 11.6	1.00	-
> 49.683	100.00	88.4 - 100.0	86.67	69.3 - 96.2	7.50	0.000
> 50.999	96.67	82.8 - 99.9	86.67	69.3 - 96.2	7.25	0.038
**> 55.764**	**96.67**	**82.8 - 99.9**	**100.00**	**88.4 - 100.0**	**-**	**0.033**
>209.969	0.00	0.0 - 11.6	100.00	88.4 - 100.0	-	1.000
